# Motor planning poststroke: impairment in vector‐coded reach plans

**DOI:** 10.14814/phy2.12650

**Published:** 2015-12-10

**Authors:** John‐Ross Rizzo, Todd E. Hudson, Andrew Abdou, Ira G. Rashbaum, Ajax E. George, Preeti Raghavan, Michael S. Landy

**Affiliations:** ^1^ Rusk RehabilitationLangone Medical CenterNew York UniversityNew YorkNew York; ^2^ Department of Psychology and Center for Neural ScienceNew York UniversityNew YorkNew York; ^3^ School of MedicineUniversity of Medicine and Dentistry of New Jersey NewarkNew Jersey; ^4^ Neuroradiology Section Department of RadiologyLangone Medical CenterNew York UniversityNew YorkNew York

**Keywords:** Motor learning, movement planning, reaching, stroke

## Abstract

Healthy individuals appear to use both vector‐coded reach plans that encode movements in terms of their desired direction and extent, and target‐coded reach plans that encode the desired endpoint position of the effector. We examined whether these vector and target reach‐planning codes are differentially affected after stroke. Participants with stroke and healthy controls made blocks of reaches that were grouped by target location (providing target‐specific practice) and by movement vector (providing vector‐specific practice). Reach accuracy was impaired in the more affected arm after stroke, but not distinguishable for target‐ versus vector‐grouped reaches. Reach velocity and acceleration were not only impaired in both the less and more affected arms poststroke, but also not distinguishable for target‐ versus vector‐grouped reaches. As previously reported in controls, target‐grouped reaches yielded isotropic (circular) error distributions and vector‐grouped reaches yielded error distributions elongated in the direction of the reach. In stroke, the pattern of variability was similar. However, the more affected arm showed less elongated error ellipses for vector‐grouped reaches compared to the less affected arm, particularly in individuals with right‐hemispheric stroke. The results suggest greater impairment to the vector‐coded movement‐planning system after stroke, and have implications for the development of personalized approaches to poststroke rehabilitation: Motor learning may be enhanced by practice that uses the preserved code or, conversely, by retraining the more impaired code to restore function.

## Introduction

Stroke is the leading cause of serious long‐term disability in the United States with over seven million survivors (Go et al. 2014). Hemiparesis is the most common motor impairment, which limits the ability to perform activities of daily living (Nakayama et al. 1994a,b). A clearer understanding of poststroke motor impairment may assist in developing therapeutic strategies to improve function. It has been shown that planning of hand trajectories during reach is impaired after stroke (Beer et al. 1999; Cirstea et al. 2003); impaired planning means an inability to predict the consequences of motor action in space and time (Beer et al. 1999; Kusoffsky et al. 2001; Takahashi and Reinkensmeyer 2003; Ketcham et al. 2007). It has also been shown that the preparation of hand posture to grasp an object based on its shape (Raghavan et al. 2010) and of fingertip forces based on object weight (Raghavan et al. 2006) is impaired poststroke. An inability to prepare appropriate motor outputs likely results from impaired sensory‐motor integration or impaired learning, which can impact poststroke re‐learning and recovery (Tseng et al. 2007). Adequate assessment and targeted treatment of planning deficits may lead to improved motor re‐learning and functional recovery.

Two movement‐planning codes for reaching have been described: a vector code that represents the direction and extent of a desired movement (Ghez et al. 1995, 1997; Gordon et al. 1995; Rossetti et al. 1995; Vindras et al. 2005), and a target code that represents the desired endpoint (Shadmehr et al. 1993; van den Dobbelsteen et al. 2001; Thaler and Todd 2009). Recent evidence has revealed that healthy, young individuals use both vector and target codes for reach planning (Hudson and Landy 2012). If two movement plans are computed using vector and target codes, the plans may be combined to produce more precise movement execution. This is similar to the performance enhancements found with perceptual estimation from multiple sensory cues (Landy et al. 1995; Hillis et al. 2002), even partially correlated cues (Oruç et al. 2003), both within (Landy and Kojima 2001) and across modalities (Hillis et al. 2002), generally by a weighted average in which a cue's weight is proportional to its reliability. Additionally, there is a near‐optimal combination of prior and current sensory information (Tassinari et al. 2006) and reference frames during motor planning. Analogously, one potential advantage of a system that makes use of two movement‐planning codes is that it can generate superior statistical performance relative to a movement‐planning system with a single code (Hudson and Landy 2012). One of these systems or the integration of their outputs may be compromised poststroke; this may have further implications for impaired sensory cue integration, as it relates to motor planning, following neurologic impairment.

Here, we test whether vector and target reach‐planning codes are differentially affected after stroke relative to healthy controls. We hypothesized that code‐specific practice should have predictable effects on mapping precision, and used this to test for the ability to use vector and target planning codes in each cohort. It is possible that injury to one side of the brain and, more specifically, area within that hemisphere after a stroke may differentially affect the vector and/or target planning codes. In fact, it has been shown that there is hemispheric asymmetry in reach planning with the left hemisphere being more dominant for planning with both the right and left hands (Schaefer et al. 2012). If it is known that movement planning is impaired relative to one or both coding systems, it may be possible to implement a training strategy that utilizes code‐specific practice to assist in restoring function.

## Materials and Methods

The study was approved by the Institutional Review Board of New York University, and informed consent was obtained.

### Participants

Twenty‐four participants were recruited from New York University/New York University Langone Medical Center and completed the study: 12 control participants (25–55 years old) and 12 stroke participants (23–71 years old; Table 1). All participants performed identical target‐grouped and vector‐grouped reaches. The protocol was shortened for the stroke participants to facilitate task completion. All stroke participants completed the experiment with their less affected arm, and nine of them also performed the reaches with their more affected arm.

**Table 1:** Clinical characteristics of participants; neuroradiologist – A. G

Participant ID	Age (years)	Sex	H/H^a^	Stroke characteristics^b^	Chronicity (years)	Fugl–Meyer score^c^
1	55	M	R/R	L MCA infarct: basal ganglia	3.1	60
2	45	M	R/L	R MCA infarct: corona radiata and basal ganglia	4.9	31
3	49	M	L/R	L MCA infarct/bleed: frontal, parietal, temporal lobes, and basal ganglia	4.8	24
4	48	F	R/L	R MCA infarct: frontal, parietal, temporal lobes, and basal ganglia	8.7	4
5	32	F	R/L	R MCA infarct: frontal, parietal lobes, and basal ganglia	7.8	49
6	44	F	R/L	R MCA infarct: frontal and parietal lobes	5.3	61
7	59	M	R/L	R MCA infarct^d^	4.3	65
8	71	F	R/R	L MCA infarct: parietal lobe + corona radiata and basal ganglia	10.5	59
9	41	M	R/L	R MCA infarct/bleed: frontal, temporal, occipital lobes, and basal ganglia	6.1	44
10	38	M	R/R	L MCA infarct: frontal, parietal, temporal lobes, and basal ganglia	7.6	28
11	54	M	R/R	L MCA infarct/bleed: frontal, temporal lobes, and basal ganglia	14.8	23
12	59	M	R/R	L MCA infarct: corona radiata, thalamus, and basal ganglia	5.3	15
Avg (SD)	49.6 (10.7)				6.9 (3.2)	38.6 (20.4)

a H/H = Handedness (as assessed by Edinburgh Handedness Inventory)/Hemiparesis Laterality (as assessed by clinician).

b Stroke subtype: lesion location obtained from MR and based on radiology reports.

c Fugl–Meyer scale (functional motor impairment tool): this score reflects the sum of the upper extremity score (out of 36) and hand/wrist score (out of 30).

d No MR available secondary to contraindication, “territory” described by cerebral vasculature.

#### Inclusion/exclusion criteria

We recruited participants with both right and left hemiparesis meeting the following criteria. The inclusion criteria were as follows: (1) age >21 years; (2) stroke >4 months old; (3) intact ocular motility; and (4) ability to complete “practice” reaches for the experiment. The exclusion criteria were as follows: (1) significant cognitive dysfunction, as defined by a score <23 on Folstein's Mini Mental Status Examination (Cockrell and Folstein 1988); (2) severe or unstable spasticity, defined as Modified Ashworth Scale (MAS) ≥2; (3) depression, as defined by the Geriatric Depression Scale score >11; (4) major disability, as defined by the modified Rankin Scale >4 (van Swieten et al. 1988); (5) previous neurological illness, complicated medical condition, or significant injury to the eye or upper limb; (6) visual–motor skill deficits, as defined by a score of <20 on the Beery Test of Visual–Motor Integration (VMI) (Brown 1977; Breen 1982; Malloy et al. 2003); (7) poor visual acuity, as defined by an acuity <20/30 OU on the Snellen chart (Tannenbaum 1971); (8) visual field impairment, as assessed by confrontation testing, if in question Goldmann and Humphrey peripheral field testing was performed (Beck et al. 1985); (9) hemispatial neglect, as defined by >6 mm of midline deviation on Schenkenberg's line bisection test (Schenkenberg et al. 1980) and >3 errors on the single‐letter cancellation test (Johnston and Diller 1986).

### Rationale

Previous work on the nature of the coding and coordinate systems used for movement planning has relied on manipulations of the movement task (Ghez et al. 2007; Thaler and Todd 2009), or the availability/precision of sensory inputs (McGuire and Sabes 2009). Sensory information can be varied by removing vision of the hand and/or target, blurring the target, allowing the target to be touched with the nonreaching hand, or altering the proprioceptive/cutaneous feedback from finger contact with the target. Here, we take a different approach: We maintain constant sensory inputs while manipulating prior movement history, thereby controlling the information used to learn the association between the movement code and motor output.

We reasoned that we could selectively improve the precision of the representation for vector‐ or target‐coded movement plans by grouping reaches described by the same displacement vector (“vector‐grouped,” e.g., performing several up‐and‐to‐the‐right movements from various starting points) or reaches to the same target location (“target‐grouped,” e.g., performing several movements ending at the same location, but with different start locations). We were interested in isolating contributions due to the precision of internal models using these two movement codes. Thus, it was crucial to eliminate any differences in sensory input or motor execution between the two conditions. Thus, we designed an experiment in which reaches required for both vector and target codes were identical, the order was simply manipulated to group them based on the aforementioned vector or target pattern.

In this experiment, controls and participants with stroke made speeded reaches (under a time constraint) on a tabletop to an array of targets displayed on a computer monitor. Repeated movements to the same target (e.g., on the upper left), where the set of movements defines several different vectors (i.e., start positions relative to a common endpoint) were made to selectively improve the representation of that target within a target code. Similarly, a group of movements with the same vector (e.g., up‐and‐to‐the‐right), each from a different start position and to a different target, was made to selectively improve the representation of that movement direction and extent within a vector code. Hudson and Landy (2012) showed that vector‐grouped practice produces reach endpoint errors forming elongated covariance ellipses oriented along the direction of the reach (i.e., the major axis is aligned with the reach direction indicating that errors were typically larger along the reach as compared to orthogonal to the reach), but target‐grouped practice produces endpoint errors described by roughly circular error ellipses (i.e., not oriented in any particular direction). We compare these same indicators of code‐specific errors in control participants to participants with stroke, by computing the ratio of the component of 2D variance along versus perpendicular to the reach direction. An unoriented (circular) ellipse will have a variance ratio indistinguishable from one, whereas an elongated ellipse will produce a variance ratio greater than one.

### Apparatus

**Figure 1:**
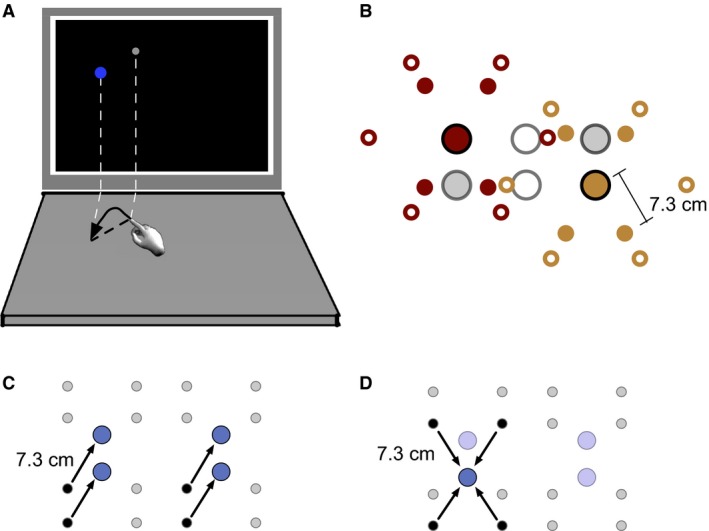
Experimental apparatus and design. (A) Schematic of experimental apparatus (not to scale). Reaches were made from point to point on a tabletop to virtual targets that were presented on the display screen. (B) Reaches were made to one of four targets arranged in a 2 × 2 grid (large circles; 3 × 2 for controls) from start positions arranged on a circle around each target (small circles, open for controls [6] and filled for stroke participants [4]). For clarity only two groups of start positions, around the upper left (red) and lower right (gold) target, are shown here. (C) Full grid of target and start positions used by stroke participants, highlighting one of the four reach vectors. (D) Full grid of target and start positions, highlighting reaches to one of the four targets. Note that the highlighted portions of C and D are used as icons in the remaining figures to indicate when data are taken from the vector‐grouped or target‐grouped conditions of the experiment.

Participants were seated 42.5 cm in front of a 21″ computer monitor, mounted to the tabletop with its center 26.5 cm above the table (Fig. 1A). The table extended 35.3 cm in front of the monitor. Fingertip positions were continuously monitored via two Optotrak 3020 cameras tracking six infrared light‐emitting diodes (IREDs) on a metal ring. The ring was worn on the right index finger by all control participants, and on either the index finger or the metacarpophalangeal joint of the index finger in stroke participants. Calibration was performed prior to each experimental session.

### Stimuli

Participants attempted to touch circular, virtual targets on the tabletop (Fig. 1A). Virtual targets were shown in corresponding locations on a computer screen (1:1 screen:actual displacement). Target radius was determined for each participant separately at the end of the practice phase to equate hit rates across participants to between 40% and 50%. Reach distance (start–target separation) was 11.75 cm in the control and 7.3 cm in the stroke group to facilitate task completion. The hand was visible below the computer screen, but all instructions were displayed on the screen and there were no visible landmarks on the featureless table. Under these conditions, our participants generally do not fixate the hands (Hudson and Landy 2012).

#### Stroke participants

Reaches were made to four targets arranged on a 2 × 2 grid (row spacing: 6.4 cm, column spacing: 13.6 cm). The target‐relative orientations of reach start positions were the four directions ±60° relative to horizontal (Fig. 1B). Target size was set, separately for each arm, such that it would have produced a 50% hit rate for practice reaches and ranged from 5.8 to 12.4 mm.

#### Control participants

Reaches were made to six targets, arranged as two rows of three (Fig. 1B; row spacing: 6.4 cm; column spacing: 6.8 cm; these were the same as for stroke participants, but with an additional column of 2 targets). Around each of the six targets were six possible start positions, positioned at 60° intervals around the corresponding target. Based on the results of practice reaches, target size was set such that it would have produced a 40% hit rate for those reaches and ranged from 2.6 to 6.6 mm.

### Procedure

Participants performed two repetitions of a single set of experimental reaches, once grouped according to reach vector and once according to reach target. Half of participants performed vector‐grouped reaches prior to target‐grouped reaches and the other half in reverse order. Participants brought the hand to the start position as cued by a visual indicator. Next, a blue dot (2 mm radius) at the target center was displayed to indicate the reach target, followed 50 msec later by a brief tone indicating that participants could begin the reach when ready. The movement was required to be completed within 300 msec of movement onset (1 sec for stroke participants); slow reaches were repeated. No feedback of the fingertip was displayed onscreen during reaches. The location of the fingertip at the end of the movement was displayed as a static red dot, along with a circular target whose radius was computed to result in a 40–50% hit rate. Targets turned blue and a pleasant auditory tone was played when reach endpoints fell within the target.

Prior to each set of experimental reaches, participants made a small number of practice reaches that had neither a target nor a vector grouping. These reaches were designed to provide familiarity with the reach procedure and time constraints, and allowed for a measurement of overall variance (to set the size of the target disk).

### Experimental reaches

All participants with stroke performed vector‐grouped and target‐grouped reaches with their less affected arm; a subset of the participants with stroke repeated the experiment with the more affected arm. Each set of grouped reaches consisted of nine repetitions of the 16 start position/target combinations (144 per grouping for a total of 288 reaches). Participants were given the identical 16 reaches in both sets; the only difference was the order in which they were given. Vector‐grouped reaches (Fig. 1C) kept the vector that defined the desired reach trajectory and distance constant for a block comprising all nine repetitions of the four reaches defined by that vector (chosen in random order), then another of the four vectors was chosen and all reaches completed, until all four vectors had been accounted for. Similarly, target‐grouped reaches (Fig. 1D) kept the target position constant for a block of 36 reaches (chosen in random order), followed by a block corresponding to a different target position, and so on for four blocks. Conditions were identical for control participants, except for the fact that they were given 36 distinct reaches (six targets and six start positions associated with each target), and completed 12 repetitions of each for each grouping for a total of 432 reaches per grouping.

### Statistical analysis

Analysis of variance was performed to examine differences between groups (stroke vs. controls) and within group (target vs. vector) on reach distance, overall duration, acceleration duration, variance, variance ratio, and standard deviation of reach direction. For example, a nested test was required for comparison of more versus less affected arms in stroke, since all more affected data were produced by participants from the less affected group. Most tests used a mixed design with factors for participants (12 participants for control and less affected arm in stroke, and 9 for the more affected arm in stroke), reach vector or target (6 targets or vectors for control, and 4 for stroke participants, averaged across either 9 or 12 repetitions each for stroke or control participants, respectively), and two for grouping (target‐ vs. vector‐grouped).

### Methodological differences between stroke and control sessions

There were several differences between the experimental reaches made by control and stroke participants. Reaches performed by stroke participants were constrained to a 1‐sec duration (vs. 300 msec) and to targets placed 7.3 cm away from the start location (vs. 11.75 cm). Furthermore, two of the six start and target locations in each half of the experiment were not given to patients and only nine (vs. 12) repetitions of each target were given, reducing the total number of reaches from 864 per session (vs. 244). These differences in methodology were all implemented to allow stroke participants to complete reaches that were as similar as possible to those made by controls, without being overly taxing or difficult for the patients to perform.

## Results

### Reach kinematics

**Figure 2:**
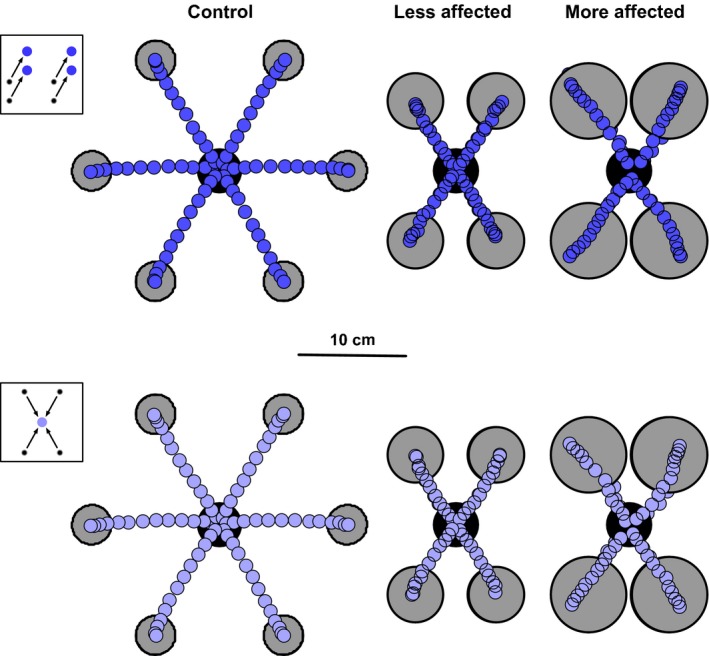
Average reach trajectories, plotted as fingertip position in the horizontal plane relative to the start position (plotted overlapping in the center) while reaching in one of the four directions (six for controls); plot symbols are equally spaced in time (i.e., 2D position is plotted as a function of time, where each plot symbol represents a fixed percentage of the total average reach time). Target circles (gray with black border) are drawn to scale to represent the average size of the targets presented.

Figure 2 shows the pooled spatial trajectories of the reaching movements for vector‐ and target‐grouped reaches made by controls and the more and less affected arms of stroke participants. Note that the trajectories of the more affected arm resulted in hypermetric reach extents, as compared to those of the less affected arm and controls (*F*_1,155_ = 38.9 and *F*_1,195_ = 14.4, *P *<* *0.05); however, the observed hypermetria did not differ between vector‐ and target‐grouped reaches (*F*_1,62_ = 0.28, *P *>* *0.05).

**Figure 3:**
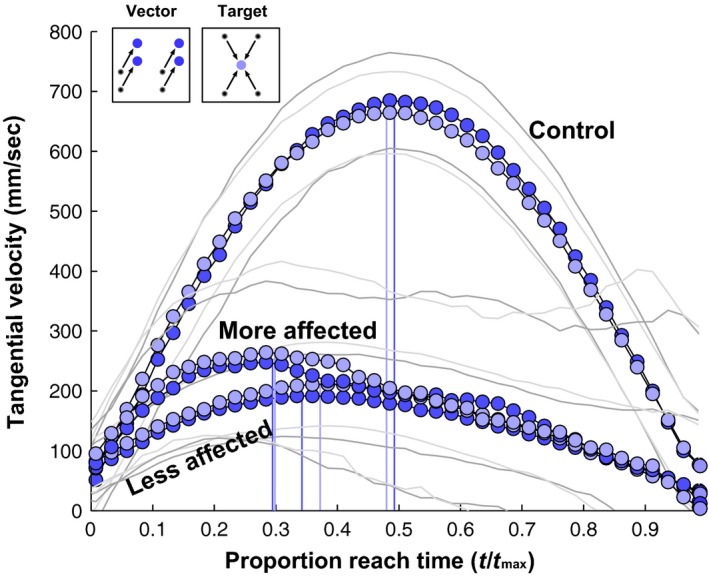
Velocity profiles plotted over the course of the reach (collapsed across participants and reach vectors and targets) as a function of the time since reach initiation (*t*) relative to reach duration (*t*_max_). Error regions indicate 95% confidence intervals. The vertical lines indicate time of peak velocity.

The mean tangential velocity, as expected, was higher in controls compared to the more and less affected arms of stroke participants, but there were no statistically significant differences between vector‐ and target‐grouped reaches within the three groups (*t*‐tests at each relative time point shown in Fig. 3, all *P *>* *0.05). The absolute reach durations were also not significantly different between vector‐ and target‐grouped reaches within any of the three groups (control: *F*_1,131_ = 0.25; less affected: *F*_1,83_ = 0.6; more affected: *F*_1,62_ = 0.23, all *P *>* *0.05).

The velocity peaked at nearly the halfway point in controls showing a bell curve with symmetric acceleration and deceleration phases. However, the velocity peaked at about 35% and 30% of the total normalized reach duration in the less and more affected arms of stroke participants, respectively, showing a shortened acceleration phase and a more prolonged deceleration phase (Fig. 3, stroke vs. control *F*_2,22_ = 30.9, *P *<* *0.05; more vs. less affected arms of stroke participants *F*_1,12_ = 2.9, *P *>* *0.05).

### Variance in reach endpoint

**Figure 4:**
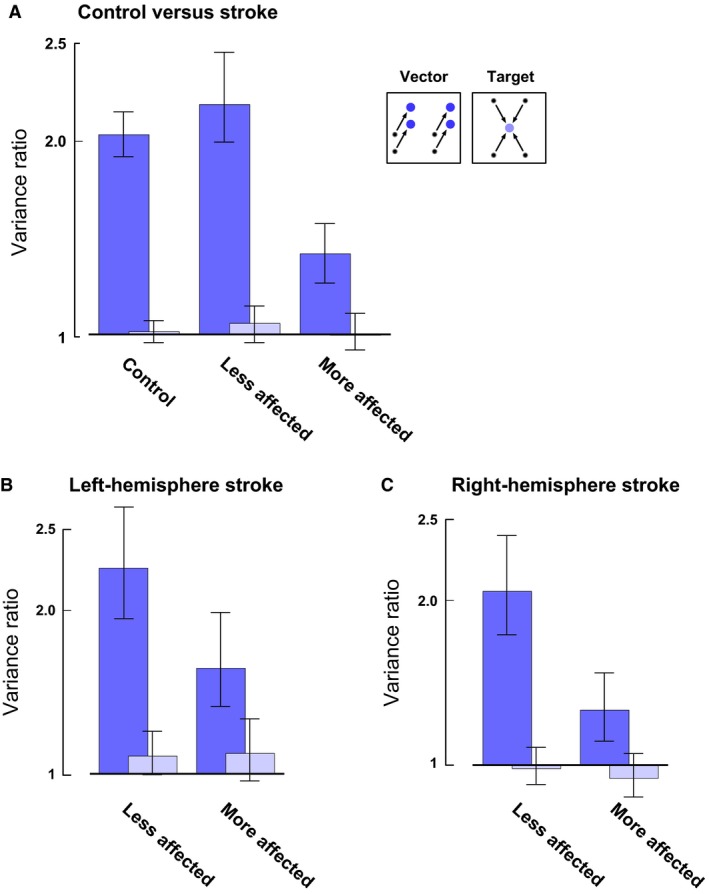
(A) Ratio of variances (parallel/perpendicular to the reach direction) for vector‐ and target‐grouped reaches. Panels (B) and (C): same as in (A), separated by side of stroke. The data were pooled from all reaches by rotating the data relative to the starting point as if all reaches were up and to the right. The same scale is used throughout so that left (*n *=* *4 for more affected; *n *=* *6 for less affected) and right (*n *=* *5 for more affected; *n *=* *6 for less affected) hemisphere stroke groups may be compared. In all panels, error bars indicate the 95% confidence interval.

To examine the pattern of variability at the endpoint, we plotted the ratio of the variance in the direction of the reach versus that perpendicular to it (Fig. 4A) and compared the shape of the error covariance between control and stroke participants. Control participants showed elongated covariance ellipses for vector‐grouped reaches; the reach variance was twice as large in the direction of the reach compared to the variance perpendicular to it (variance ratios significantly greater than unity, *F*_1,59_ = 49.5, *P *<* *0.05), whereas the error covariance was circular for target‐grouped reaches (variance ratios indistinguishable from unity, *F*_1,59_ = 0.9, *P *>* *0.1). This dichotomy in covariance ratios between vector‐ and target‐grouped reaches also held true for the less affected (vector: *F*_1,35_ = 43.3, *P *<* *0.05; target: *F*_1,35_ = 1.9, *P *>* *0.1) and more affected (vector: *F*_1,26_ = 15.8, *P *<* *0.05; target: *F*_1,26_ = 10.6, *P *>* *0.1) arms of participants with stroke. However, the vector‐grouped variance ratios in the more affected arm were significantly smaller than those in the less affected arm (*F*_1,29_ = 12.7, *P *<* *0.05).

We then examined the covariance ratio of the more affected arm as a function of the side of hemiparesis to determine if coding is lateralized to a hemisphere. The vector system was mildly impaired in the more affected arm of participants with left‐hemisphere stroke, presenting with right hemiparesis (Fig. 4B more vs. less affected vector ratios, *F*_1,13_ = 4.5, *P *>* *0.05), but it was more impaired (i.e., a larger decrement in the vector ratios for the more affected arm) in participants with right hemisphere stroke, presenting with left hemiparesis (Fig. 4C, vector‐grouped ratios are significantly reduced in more relative to less affected arms, *F*_1,15_ = 7.6, *P *<* *0.05). Note that the vector‐ and target‐grouped variance ratios for the less affected arm in both left (vector: *F*_1,17_ = 21.6, *P *<* *0.05; target: *F*_1,17_ = 0.41, *P *>* *0.1) and right hemisphere (vector: *F*_1,17_ = 21.3, *P *<* *0.05; target: *F*_1,17_ = 0.26, *P *>* *0.1) stroke groups showed the expected patterns as seen in controls.

It is possible that increased motor noise toward the ends of reaches (“late noise” added during reach execution) could affect reach endpoints in patients with right hemisphere stroke more than those with left hemisphere stroke. Such a differential increase in overall endpoint variance would reduce the degree of anisotropy in the right hemisphere stroke group compared to those with left hemisphere stroke, thus masking the elongation of error ellipses in the former group, but not the latter. However, there was no statistical difference in the variance of the trajectory endpoint between left and right hemisphere stroke for vector‐grouped reaches on the more affected side (*F*_1,27_ = 0.38, *P *>* *0.1), suggesting that late noise cannot explain the change in vector ratios between right versus left hemisphere stroke.

As expected, reaches made with the more affected arm were noisier than those made with the less affected arm. To see if this increased variability had any directional preference, we analyzed the standard deviations of reach direction for each of the four target directions in the stroke‐specific reaching paradigm. However, we found no differences in directional error between left and right hemisphere stroke.

## Discussion

Hudson and Landy (2012) demonstrated the existence of two movement‐planning subsystems, a vector‐coded and a target‐coded system, via practice‐mediated changes in the precision of sensory‐motor mappings, while keeping the task, biomechanics, and sensory inputs nearly constant. Those experiments involved natural, unperturbed reaches in support of the theory that multiple coding systems for reach plans are the norm during everyday movements in healthy controls. Here, we provide evidence that a particular subsystem, in this case vector, may be more vulnerable post‐MCA stroke based on our analysis of the kinematics and variability during point‐to‐point, visually guided reaching.

### Deficits in reach accuracy and velocity after stroke

We observed significant hypermetria for reaches with the more affected arm during both vector‐grouped and target‐grouped reaches. Hypermetric reaches overshoot the target, and therefore represent a persistent error in reach gain. This may represent a problem in gain planning. These reach accuracy deficits were similar for vector‐ and target‐grouped reaches, suggesting that the type of practice did not modify them.

Peak velocity occurred earlier in the reach with the more and less affected arms in stroke participants relative to controls, although relative time to peak velocity was not different in vector‐coded versus target‐coded conditions. The earlier peak velocity suggests a deficit in feedforward control of movement, and a greater dependence on feedback control for accurate reaches (Raghavan et al. 2006, 2010; Tseng et al. 2007). It is interesting, but not unexpected, that both the less and more affected arms showed deficits in feedforward control. Deficits in motor planning have been demonstrated in the less affected hand after stroke (Smutok et al. 1989; Haaland et al. 2004, 2009; Yarosh et al. 2004; Noskin et al. 2008; Seo et al. 2009; Lindberg et al. 2012), and have typically been noted in patients with severe hemiplegia where such deficits cannot be assessed in the more affected hand. Less affected arm deficits may be due to damage to specific areas of the brain that control aspects of movement with both hands (Shabbott and Sainburg 2008; Schaefer et al. 2012; Coelho et al. 2013; Mutha et al. 2013). It has been suggested that the left hemisphere is specialized for feedforward control (planning) of motor output, whereas the right hemisphere is specialized for feedback control to update ongoing action, including stopping at the target (Sarlegna and Sainburg 2009; Schaefer et al. 2012; Timmis and Pardhan 2012).

### Endpoint variability of reaching movements poststroke

One signature of the vector‐based system is anisotropy of endpoint variance (van Beers et al. 2004). In control participants and in the less affected arm of participants with stroke, the variance along the direction of the reach was double the variance orthogonal to that direction, as has been shown before (Hudson and Landy 2012). Interestingly, this variance ratio was reduced for the more affected arm in participants with stroke, particularly for those with right hemisphere strokes. Taken together with previous suggestions of right hemisphere specialization for feedback control (Schaefer et al. 2012), our data suggest that vector‐based planning may depend more on and be optimized by feedback. While the sample size was too small to make broad generalizations regarding specific training strategies for individuals with right or left hemisphere stroke, this finding opens up avenues for exploring the types of feedback strategies that might be useful to enhance vector‐based practice after stroke.

Impairment of movements with the hemiparetic upper limb is known to be directionally dependent (Beer et al. 2000). Movements are often biased in specific directions (Meulenbroek and Thomassen 1991), which is thought to occur due to abnormal multijoint coordination at the motor‐command level (Hollerbach and Flash 1982), and may be a consequence of typical poststroke synergy patterns (Dipietro et al. 2007). We explored if the vector code was particularly impaired in specific directions, but found no differences in the standard deviations of reach trajectories in the vector and target conditions.

In contrast to our analysis of the vector‐coded system, we found little evidence of impairment to the target‐coded planning system after stroke. If the target code were damaged, we would expect target‐grouped reaches to be dominated by the vector code, resulting in anisotropic variance ratios. Yet, target‐grouped variance ratios were near unity for the less affected arm and for the more affected arm of patients with stroke independent of the side of injury. Thus, our results demonstrate a specific deficit in vector‐coded movement plans in participants with stroke.

### Implications for rehabilitation strategies

The current practice of poststroke upper limb rehabilitation advocates for task‐specific practice (Bayona et al. 2005). Patients may practice reaching tasks in several ways: the reaches may have a common endpoint from various starting locations in space (target practice), or they may have a common vector, maintaining the same direction and extent, but with differing endpoints (vector practice), or there may be a combination of the two types. It may be that individuals with impaired vector‐based movement planning are unable to learn from vector practice. In this case, they may need to use target practice to improve their reach accuracy. Alternatively, individuals with impaired vector‐based movement planning may require a greater amount of feedback during vector‐specific practice. It is also possible that specific types of visual or proprioceptive feedback would enhance vector‐based practice relative to target‐based practice (Cho et al. 2014; Kim et al. 2015).

In summary, there are many types of practice strategies implemented for retraining upper limb movements during stroke rehabilitation (Dobkin 2008; Hubbard et al. 2009), however the use of standardized target versus vector practice is not typical, nor is the assessment of code‐specific deficits. Our results suggest that understanding the nature and degree of deficits in movement coding plans may help determine specific practice strategies needed to optimize motor re‐learning following stroke.

## Conclusion

Taken together, our results further confirm the existence of movement‐planning deficits after stroke, in addition to movement execution deficits in both more and less affected arms after stroke. While planning deficits may occur in either the vector‐coded movement‐planning system that specifies reach direction and extent and/or the target‐coded movement‐planning system that specifies reach location in space, we found that the vector‐coded movement‐planning system is impaired in individuals with MCA‐distribution strokes, particularly those with right brain damage. Our findings have potential implications for selecting the appropriate practice strategies to optimize motor learning. These findings should be tested further in larger cohorts with improved lesion mapping for greater neuroanatomic specificity. Ultimately, these code‐specific approaches may pave the way for more individualized and tailored rehabilitation after stroke.

## Conflict of Interest

None declared.
